# Biologic Agents for Periodontal Regeneration and Implant Site Development

**DOI:** 10.1155/2015/957518

**Published:** 2015-10-05

**Authors:** Fernando Suárez-López del Amo, Alberto Monje, Miguel Padial-Molina, ZhiHui Tang, Hom-Lay Wang

**Affiliations:** ^1^Department of Periodontics and Oral Medicine, University of Michigan School of Dentistry, Ann Arbor, MI 48109, USA; ^2^Department of Oral Surgery and Implant Dentistry, University of Granada, Granada, Spain; ^3^2nd Clinical Division, Peking University School of Stomatology, Beijing, China

## Abstract

The advancement of molecular mediators or biologic agents has increased tremendously during the last decade in periodontology and dental implantology. Implant site development and reconstruction of the lost periodontium represent main fields in which these molecular mediators have been employed and investigated. Different growth factors trigger different reactions in the tissues of the periodontium at various cellular levels. Proliferation, migration, and differentiation constitute the main target areas of these molecular mediators. It was the purpose of this comprehensive review to describe the origin and rationale, evidence, and the most current understanding of the following biologic agents: Recombinant Human Platelet-Derived Growth Factor-BB (rhPDGF-BB), Enamel Matrix Derivate (EMD), Platelet-Rich Plasma (PRP) and Platelet-Rich Fibrin (PRF), Recombinant Human Fibroblast Growth Factor-2 (rhFGF-2), Bone Morphogenic Proteins (BMPs, BMP-2 and BMP-7), Teriparatide PTH, and Growth Differential Factor-5 (GDF-5).

## 1. Introduction

Multiple factors and/or conditions such as periodontal diseases, traumatisms, congenital abnormalities, and tumors may result in significant loss of the periodontium and surrounding structures. If no treatment is provided then the risk for extensive bone defects increases and this may eventually compromise the ability of maintaining the dentition or even the overall bony structure for future prosthetic rehabilitation. Hence, in these scenarios, not only periodontal treatment is required but also regain of the lost structures is recommended. Regeneration of the lost periodontium with exact same structure has been the ideal goal in periodontal therapy for long time [1]. However, the reestablishment of the periodontium's original form, shape, properties, and function remains a clinical challenge [2]. By definition, periodontal regeneration must achieve the regeneration of the alveolar bone, cementum, and periodontal ligament and also promote an adequate sealing by the gingival tissue. To do so, a temporal sequence and specific spatial distribution of the cells and signaling molecules involved in this particular healing process have to be followed [3].

The specific mechanisms and events necessary for periodontal regeneration to occur are not yet fully understood. It is known however that specific cells must first attach to the substrate before migrating and proliferating to the healing area supported by the fibrin coagulum and attracted by soluble factors. Once in the area, those cells will provide the cellular and molecular machinery needed to clean the area and initiate the growth of the new tissue. As it progresses, extracellular matrix (ECM) and matrix-cellular proteins will secure the area so that differentiated populations can act to, ultimately, establish a functional tissue with the appropriate turnover stimulated by function and supported by blood supply [4].

Since its development in 1980s [5–7], guided tissue regeneration (GTR) has been extensively studied. With the vast amount of clinical and preclinical studies, it has been demonstrated that contained defects, such as 3-walled intrabony and class two furcations, can predictably be regenerated with the employment of multiple grafting materials and/or barrier membranes [8]. However, when significant tissue regeneration is needed or in the presence of locally and/or systemically compromised scenarios (i.e., restricted blood supply due to systemic disorder or uncontained defect), the application of grafting materials, barrier membranes, and/or biologic agents has to be carefully selected to promote predictable and sufficient quantity and quality of regenerated tissue [8]. Therefore, not only scaffolds but also ability to inhibit invasion of certain undesired cells to the area is needed. Nonetheless, scaffolds by themselves usually fail to promote the adequate healing induction and conduction needed for the repair process to occur. When it does, this healing cannot be considered a true regeneration because it usually promotes bone regeneration failing at reconstructing other components of the periodontium. The application of biologic agents emerges to compensate for such drawbacks by mimicking, inducing, and regulating the activity of the natural events that happen in the healing area with the purpose of promoting the regeneration of the tissue. Biologic agents are substances made from a living organism or its products used in the prevention, diagnosis, or treatment of a disease. In this sense, the utilization of such biomaterials should ideally result in faster healing and improved regenerative outcomes. A wide variety of biomolecules, compounds, and purified recombinant agents have been proposed (Table 1). Therefore, the purpose of this review was to comprehensively review each particular biologic agent and provide the rationale, evidence, and current understanding of the effect of these materials upon periodontal regeneration.

## 2. Recombinant Human Platelet-Derived Growth Factor-BB (rhPDGF-BB)

### 2.1. Rationale

rhPDGF-BB represents one of the most widely studied growth factors in the field of periodontology. Its efficacy has been demonstrated in both hard and soft tissue regeneration during the last decades. Specifically, PDGF-BB is involved in wound healing stimulating the potential for regeneration of the periodontal tissues [9]. Since it was firstly investigated in periodontology [10], multiple studies have then focused on a better understanding of the mechanism of action as well as its therapeutic potential. Today, three different forms of PDGF are known: PDGF-AA, PDGF-AB, and PDGF-BB. After hard or soft tissue injury, PDGF is released by blood platelets binding to specific cell surface receptors. As a consequence, enhancement of wound healing process by means of chemotaxis and mitogenesis occurs [11, 12]. Of particular importance for the field of periodontology is GEM 21S, (Osteohealth, Boston, MA). Its use has been extensively investigated in preclinical and clinical studies including both animal and human subjects. This product uses *β*-tricalcium phosphate (*β*-TCP) as a carrier of a highly purified rhPDGF-BB, providing physical structural support and space maintenance.

### 2.2. Evidence/Indications

Numerous animal and human studies have demonstrated its potential for periodontal and peri-implant regeneration [10, 13–16]. Briefly, animal studies have demonstrated that PDGF is able to promote bone, cementum, and periodontal ligament regeneration in periodontal defects in dogs [10]. Additionally, further studies have investigated the potential of PDGF around dental implants concluding that the application of PDGF/IGF resulted in a significant increase in percentage of bone fill and bone-to-implant contact [14]. Similarly, animal studies conducted by Simion and coworkers [17] and Schwarz and colleagues [18] investigated the potential of PDGF for both vertical bone regeneration and horizontal bone regeneration, respectively. Both studies have shown greater bone regeneration when PDGF was used in combination with the grafting material compared to using the grafting material alone. Interestingly, in the above-mentioned studies, Simion et al. tested the effectiveness of three different treatment groups for vertical bone augmentation: (1) deproteinized bovine blocks and collagen barrier, (2) deproteinized bovine blocks with rhPDGF-BB, and (3) deproteinized bovine blocks with rhPDGF-BB and collagen membrane. Histological and radiographical analysis demonstrated greater bone formation when rhPDGF-BB was utilized without collagen membrane, suggesting that membranes block the migration of bone forming cells from periosteum into the area of interest.

On the other hand, human studies have corroborated the promising potential shown in both* in vitro* and animal studies [16, 19, 20]. Although FDA only approved PDGF for treatment of periodontal related defects (e.g., intrabony defects, furcations, and gingival recessions defect), the effectiveness of this biologic agent for implant related approaches such as vertical and horizontal bone augmentation [17], sinus augmentation procedures [21], and ridge preservation procedures [22] has also been widely studied. Overall, results show greater bone formation, reduced healing times, and enhancement in the regeneration process when compared to control groups.

### 2.3. Current Understanding

PDFG has been shown to promote fibroblast, cementoblast, and osteoblast migration and proliferation into the surgical area. Currently, this agent is being tested in multiple fields of periodontology from recession coverage to vertical bone augmentation and the reconstruction of peri-implant defects. Research and clinical experience have shown its potential in improving periodontal regeneration in a variety of clinical scenarios. Nonetheless, longer and larger studies are still needed to further verify the long-term effect of PDGF.

## 3. Enamel Matrix Derivate (EMD)

### 3.1. Rationale

Enamel Matrix Derivate (EMD) is composed of different enamel related proteins, being mainly amelogenin (90%). It also contains proteins such as enamelin, tufflin, and ameloblastin, among others [23]. Embryologically, during root development, enamel matrix proteins are secreted by Hertwig's epithelial root sheath, with cementogenesis being its main function [24]. Although these proteins have shown favorable outcomes in periodontal regeneration, resulting in new bone formation, PDL, and cement [25], the exact mechanism of action remains unclear. Of particular importance in periodontology is the commercially available product (Emdogain, Institut Straumann AG, Basel, Switzerland). This product is extracted from developing porcine tooth buds. Recently, it has been demonstrated to induce the proliferation of gingival mesenchymal stem cells and enhance their osteogenic differentiation* in vitro* [26]. In an effort to elucidate its mechanism of action, Schwartz and colleagues demonstrated that EMD stimulates proliferation of preosteoblasts and differentiation of osteoblast-like cells as well as proliferation and differentiation of normal osteoblast [27]. In addition, researchers trying to elucidate its mechanism of action have recently investigated the possible effect of this product upon gene expression [28].

### 3.2. Evidence/Indications

The utilization of Emdogain has been studied in different fields of periodontology, being mainly intrabony and furcation defects as well as gingival recession coverage. When examining the efficacy of EMD for the treatment of intrabony defects, the largest study performed so far concluded that EMD provides beneficial effect in terms of CAL gain and probing depths reduction when compared to open flap debridement alone [29]. However, when compared with GTR, results from recent systematic reviews found no difference between the two [30]. On the other hand, for noncontained intrabony defects, it seems to be a benefit of GTR over EMD alone [31]. Similarly, for the treatment of class II furcation defects, EMD has been employed either alone or in combination with a variety of different grafting materials reporting different grade of success [32–34]. With regard to the treatment of recession defects, EMD has been employed with a great variety of techniques. In addition, most of the studies agree in the superiority of CAF + CTG in combination with EMD when compared to CAF alone [35, 36]. However, when compared with CAF + CTG, the results reported in the largest available study have found no differences [37]. All in all, a systematic review from the AAP Regeneration Workshop recently concluded that although all procedures aiming at recession coverage can provide significant reduction in both recession depth and CAL gain, the utilization of CTG with different techniques provides the best outcomes in terms of percentages of root coverage and increase of keratinized tissue [38].

The utilization of EMD in different areas of the implant field such as sinus augmentation and treatment of peri-implantitis defects is currently being investigated [19, 39].

### 3.3. Current Understanding

At this moment, EMD has been demonstrated to promote periodontal regeneration to a certain degree although its true effect remains to be determined. In addition, EMD has been shown to influence different genes expressed during bone remodeling (bone resorption and formation) promoting an anabolic effect [28] as well as enhancing osteoblast differentiation on titanium surfaces [40]. Future studies testing the efficacy of this material promoting guided bone regeneration (GBR) and treatment of peri-implant defects are needed. Researches are being conducted nowadays, trying to elucidate the exact mechanism of action behind the effectiveness of EMD.

## 4. Platelet-Rich Plasma (PRP), Platelet-Rich Fibrin (PRF), and Leukocyte- and Platelet-Rich Fibrin (L-PRF)

### 4.1. Rationale

Derived from megakaryocytes, platelets are small irregular anucleated cells with a diameter of 2 to 4 micrometers. Platelet average life span ranges from 8 to 12 days and the normal platelets count is defined between 150,000 and 400,000 platelets/microliter. Their key role in hemostasis and being a natural source of growth factor make platelets a component of paramount importance during wound healing. Depending on the processing technique, different types of platelets concentrates have been described including but not limited to Platelet-Rich Plasma (PRP), Pure Platelet-Rich Plasma (P-PRP), Leukocyte- and Platelet-Rich Plasma (L-PRP), Platelet-Rich Fibrin (PRF), and Leukocyte- and Platelet-Rich Fibrin (L-PRF). The potential of these substances as a biologic agent in periodontology relies on the growth factors stored within platelet alpha granules containing platelet-derived growth factor (PDGF), vascular endothelial growth factor (VEGF), insulin-like growth factor (IGF), platelet-derived angiogenic factor, and transforming growth factor-beta (TGF-*β*) [41].

### 4.2. Evidence/Indications

PRP, PRFG, and L-PRF have been tested as a substitute for connective tissue [42], as a grafting material in sinus augmentations [43], and as a barrier membrane for periodontal regeneration [44, 45], among others. Nonetheless, the effectiveness of the different concentrates is difficult to elucidate due to the great variability in study designs, different regimens of materials used (graft, membrane, or combination), surgical techniques, and so forth. Nonetheless, it has been shown to be effective in different clinical scenarios [42, 43]. However, the results may not be superior when compared to traditional CTG for recession coverage [38] or traditional GTR for periodontal regeneration [45]. On the other hand, the employment of platelet concentrates in implant and extensive bone grafting procedures remains to be determined.

### 4.3. Current Understanding

At this moment, platelet-derived concentrates have been shown to enhance soft tissue healing which indirectly can create a better environment for bone growth. However, several different concentrate products are currently being promoted without total understanding of the ideal component or concentration for the material to have the best outcome. Some have discussed the mesh that is created by fibrin as a key component of PRP, probably responsible for a great part of its properties [46]. Studies in the field are highly recommended.

## 5. Recombinant Human Fibroblast Growth Factor-2 (rhFGF-2)

### 5.1. Rationale

Discovered in 1970s as a protein inducing proliferative activity in fibroblasts, FGF-2 belongs to an extensive family with more than 20 members with similar characteristics [47]. Among all the FGFs family, FGF-2 is the most extensively studied in regenerative medicine and periodontal tissue regeneration. This protein has been studied in medicine for treatment of ulcers and bone fractures due to its potential to facilitate revascularization [47]. Additionally,* in vivo* studies have shown that FGF-2 promotes proliferation of osteoblast accelerating bone formation [48, 49]. Further animal studies have confirmed that local application of FGF-2 significantly enhances periodontal regeneration compared to control sites [50, 51]. Among its effects, FGF-2 has been shown to promote endothelial cell proliferation [52]. In addition, FGF-2 possesses a potent angiogenic and mitogenic activity on mesenchymal cells within PDL [53].

### 5.2. Evidence/Indications

FGF-2 has not been extensively studied yet in the field of periodontics. Nonetheless, several animal studies have shown that this growth factor is effective in enhancing the periodontal regeneration process for class II furcation defects in animal models [50]. For periodontal regeneration in humans, although histological results have not been yet investigated, FGF-2 has been shown to significantly improve the percentage of bone fill compared to vehicle alone [54]. In the largest available study so far comparing different concentrations of FGF-2 and a vehicle, results showed significant superiority of percentage of bone fill in FGF-2 treated sites at 36 weeks [55]. Consequently, recent systematic reviews have concluded that although scarce available evidence suggests that FGF-2 significantly improves defect fill but it does not have an effect on clinical attachment level gain [56].

### 5.3. Current Understanding

At this moment, the effect of FGF-2 remains to be determined. Future studies should continue focusing on exploring its efficacy, safety, and proper dosage for FGF-2 to be effective in different clinical scenarios.

## 6. Bone Morphogenic Proteins (BMPs, BMP-2 and BMP-7)

### 6.1. Rationale

BMPs are members of the transforming growth factor-beta (TGF-*β*) superfamily which have demonstrated high osteoinductive potential [57]. In the periodontics arena, both BMP-2 and BMP-7 stimulate PDL cell differentiation into osteoblasts and increase expression of mineralized tissue markers [58, 59]. Moreover, BMPs have been demonstrated to downregulate the mineralization process of cementoblasts. Additionally, their employment in the implant field relies on BMPs having the properties to modulate bone formation, contour, and density through endochondral formation [60] and bone formation by autoinduction [61]. Induction and maintenance of bone formation by the TGF-*β* family of proteins occur in a synergistic and synchronous way [61] with a wide variety of different proteins acting in the process [62].

### 6.2. Evidence/Indications

Its application for periodontal regeneration led to its use in implant site development (i.e., ridge preservation and sinus augmentation). It has to be noted that, when aiming at the reconstruction of the periodontium, although successful regeneration has been achieved in intrabony and furcation defects [63], complications such as ankylosis and root resorption were described [64].

rhBMP-2 (Infuse Bone Graft by Medtronic Inc.) has recently been approved by the FDA as an alternative to autologous bone grafting for sinus augmentation and ridge preservation procedures owing to its osteoinductive potential [65]. Clinical and histologic outcomes obtained for sinus augmentation showed similar pattern to autogenous bone in terms of bone quality and quantity [66]. Nonetheless, if rhBMP-2 is grafted in combination with autologous bone it seems to further increase cell activity, osteoid lines, and vascular supply, which may lead to more predictable results [66, 67]. On the other hand, Kao et al. showed that when blending rhBMP-2 with bovine-derived xenogeneic graft (Bio-Oss, Geistlich Pharma AG, Wolhusen, Switzerland) a detrimental effect occurs by means of cellular behavior and vital bone formation. This fact might be explained due to the enhancement of osteoclast differentiation by the adjustment of RANKL [68]. Nonetheless, multiple studies have demonstrated the safety of BMP-2 by lacking of immune response [66–69].

Additionally, rhBMP-2 has been studied for alveolar ridge augmentation/preservation. Although* in vivo* data is very limited, it was found to be successful in preventing socket from collapse by minimizing the horizontal bone resorption [70–72]. In this study, it was also demonstrated that the dose-dependent effect of the protein of 1.5 mg/mL shows better results than 0.75 mg/mL. It has also been shown to improve the results of sinus bone augmentation when used alone [73]. In contrast, Kao et al. reported detrimental effects when adding it into bovine-derived deproteinized bone [68]. It seems that its use is related with moderate signs of local inflammation that ultimately can jeopardize graft stability by disrupting primary wound closure. Therefore, although rhBMP-2 appears to be a promising alternative to autogenous bone grafts for alveolar ridge/maxillary sinus augmentation, dose and carrier optimization are being profoundly studied to expand its efficacy, use, and clinical application [74].

BMP-7 (Osigraft) (Osteogenic Protein-1 [OP-1]) has been only studied for sinus augmentation. van der Bergh et al. pioneered its investigation reporting a small sample case series of this growth factor embedded into a collagen sponge compared to autogenous graft. While in two subjects bone-like tissue was found, the third showed a cyst like granular tissue mass without purulent content [75]. Hence, no robust conclusion with regard to safety and efficacy could be reached. Corinaldesi et al. compared the use of rhBMP-7 + deproteinized bone (0.5 g) versus deproteinized bone alone (2 g). Results demonstrated no differences in terms of bone gain. Interestingly, newly formed bone was statistically greater for the control group (19.9% versus 6.6%) 4 months after grafting [76]. Future studies are needed to figure out the true effect of this specific combination regimen for bone regeneration.

### 6.3. Current Understanding

At this moment, BMP-2 represents a very promising alternative to the so-called “gold standard” autogenous bone. However, more studies are still needed to figure out the best carrier as well as to compare its effectiveness to the popular allogenic human allograft. The combination usage of BMP with bovine-derived deproteinized should be carefully evaluated. In any case, the induction of bone formation by the TGF-*β* proteins is a cost-effective clinical strategy according to the most recently published data [61].

## 7. Teriparatide

### 7.1. Rationale

Teriparatide consists of parathyroid hormone's (PTH) first 34 amino acids. It has been shown to influence PDL cell survival and to cause osteoblast-like behavior with increased osteoprotegerin expression [77–79]. As such, the FDA approved it for the treatment of osteoporosis. Due to its therapeutic potential, it has been further used in craniofacial regeneration. In periodontology and implantology, its effect has been tested* in vivo* in animal models showing bone formation in extraction sockets in addition to its effectiveness in three-dimensional preservation of the alveolar bone [80–82].

### 7.2. Evidence/Indications

It has been shown that, with daily injection of Teriparatide PTH and vitamin D supplement, a positive treatment outcome (gain clinical attachment levels) was achieved during the surgical treatment of periodontal intrabony defect [83]. Furthermore, this positive outcome was positively correlated with baseline levels of 1,25-dihydroxyvitamin D3. Kuchler et al. in a feasibility study found that after a healing period of 9 weeks with daily injection of Teriparatide (20 *μ*g for 28 days), bone-to-implant contact was indeed higher in the periosteal and medullary compartment but not the cortical compartment for the treated group [84]. Nonetheless, bone-volume-per-tissue-volume did not deviate statistically (was 17.6% versus 15.4% in the control group).

### 7.3. Current Understanding

At this moment, injected Teriparatide PTH showed some positive short-term effect. However, it does require more long-term studies with larger sample size. Additionally, delivery route may draw a more difficult acceptance by patients.

## 8. Growth Differential Factor-5 (GDF-5)

### 8.1. Rationale

GDF-5 is another member of the TFG-*β* superfamily. For periodontal regeneration, it has been shown, both* in vitro* and* in vivo*, to stimulate PDL cell proliferation, osteoblast differentiation (early stages), and extracellular matrix synthesis by both cell types [85]. For implant site development, GDF-5 has been demonstrated* in vivo* to induce bone in ectopic muscle pouches to improve mineralized tissue formation [86].

### 8.2. Evidence/Indications

GDF-5 has been approved by FDA for periodontal regeneration, alveolar bone regeneration, and sinus augmentation. For periodontal regeneration, it has been demonstrated to improve clinical attachment level gain (although not reaching statistical difference) [87]. GDF-5 has shown similar behavior compared to autologous graft (28% versus 32%, resp.) when bone formation was assessed at 4 months [88]. Interestingly, higher amount of bone augmentation was found in the composite group of GDF-5 + *β*-TCP. In addition, it has been shown that higher dosage (800 *μ*g) achieved higher amount of bone formation than lower dosage (400 *μ*g) after 12 weeks in the sinus cavity [89]. Nonetheless for sinus augmentation information about its effectiveness is still limited. For lateral ridge augmentation, block grafts coated with rhGDF-5 achieved similar increase of mineralized tissue when compared to block grafts impregnated with rhBMP-2. However, when evaluating graft using particulated bone, sites coated with rhGDF-5 had a significantly lower mineralized tissue formation at 8 weeks when compared to grafts coated with rhBMP-2. Moreover, nonmineralized tissue for both groups revealed high signs of osteocalcin antigen reactivity [90]. Based on these findings, rhGDF-5 has been shown to possess the potential to enhance bone formation; however, the outcome may vary with different carrier [91].

### 8.3. Current Understanding

At this moment, GDF-5 is a very promising growth factor for craniofacial regeneration due to its osteoinductive potential. However, data is still limited to prove its efficacy and safety in human. Phase II and III clinical trials are now being carried out to investigate its true effect in humans. Furthermore, the ideal carrier for delivering this promising biologic agent remains to be explored.

## 9. Other Transcription Factors and Regulators

In addition to the previously mentioned biologic agents, other critical transcription factors and regulators of osteogenesis as well as matricellular proteins may become of high interest in periodontal tissue engineering. Initial studies are being conducted on the use of* Runx2*, Osterix (Osx), LIM domain mineralization protein (LMP) [92], and periostin [93, 94], among others, with the purpose of regenerating the tooth-supporting apparatus. Although at a very early stage of these development, most of these molecules have shown promising results, such as higher new bone formation, promoted human PDL cells osteogenesis, and upregulation of alkaline phosphatase, bone sialoprotein, and BMP2,* in vivo* [95, 96].

## 10. Conclusions

The term biologic agent encompasses a variety of different growth factors and/or signaling molecules with different origin, mechanisms of action, as well as different targeted tissues and/or cells in the periodontium. In depth understanding of their mechanism of action and indications of usage is of paramount importance. While some agents are still on their infancy, others have obtained FDA approval for different clinical procedures. Although these biologics proved to be beneficial in a variety of aspects of periodontal regeneration and bone augmentation procedures, basic principles of surgery, proper patient, and/or site selection remain to be essential for predictable clinical outcomes. More investigation is required to further understand these biologic agents, providing more information with regard to long-term effects, proper carrier, and ideal concentration/dosage, among other confounding factors.

## Figures and Tables

**(a):** 

Agent	rhPDGF-BB	EMD	PRP/PRF	FGF-2
Origin	Blood platelets	Hertwig's epithelial root sheath	Platelet alpha granules	Fibroblast growth factors family

Composition	Protein	90% Amelogenin	PDGF, I-LGF, VEGF, TGF-*β*	Protein

MOA	Mainly chemotaxis and mitogenesis	Precise MOA still unknown	Combination of different MOA of different growth factors contained with the platelet concentrates	Proliferation PDL cells Migration PDL cells Differentiation PDL cells ECM production

Indications/common uses	(i) Intrabony defects (ii) Furcations (iii) Gingival recession defects (iv) Often used in combination with allograft or xenograft	(i) Intrabony defect (ii) Class II furcation defects (iii) Recession coverage procedures	(i) Recession coverage procedures (ii) Barrier membrane	(i) Peri-implant defects (ii) Intrabony defects

FDA approval	(i) Intrabony defects (ii) Furcations (iii) Gingival recession	(i) Intrabony defects (ii) Optimize tissue height in esthetic zone	Not regulated	Not yet approved

Manufacturer	GEM 21S (Osteohealth)	Emdogain (Straumann)	Multiple machines for platelet concentrates fabrications are available	Not yet commercially available

**(b):** 

Agent	BMP-2	BMP-7	GDF-5	Teriparatide
Origin	Recombinant DNA biotechnology using mammalian cells	Recombinant DNA biotechnology using mammalian cells	Recombinant DNA process using bacterial expression followed by *in vitro* refolding	Recombinant DNA

Composition	Bone Morphogenic Protein-2	Bone Morphogenic Protein-7	Growth Differential Factor-5	Parathyroid hormone's (PTH) first 34 amino acids

MOA	Increased proliferation, mineralization, and expression of alkaline phosphatase and osteocalcin	Increased proliferation, mineralization, and expression of alkaline phosphatase and osteocalcin	Increased early differentiation and matrix production	Modify proliferation of mineralized markers

Indications/common uses	(i) Systemic or anatomic condition where successful bone regeneration cannot be achieved with conventional grafts (ii) No with demineralized bovine bone	(i) Systemic or anatomic condition where successful bone regeneration cannot be achieved with conventional grafts (ii) No with demineralized bovine bone	Systemic or anatomic condition where successful bone regeneration cannot be achieved with conventional grafts	Bone metabolism disease that can jeopardize implant stability and osseointegration process

FDA approval	Sinus augmentation Socket preservation	Sinus augmentation Socket preservation	Sinus augmentation Socket preservation	—

Manufacturer	Infuse Bone Graft (Medtronic Inc.)	Osigraft (Stryker Biotech Inc.)	Scil Technology Inc.	Forteo (Eli Lilly Inc.)

MOA: mechanism of action; FDA: Food and Drug Administration.

## References

[1] Polimeni G., Xiropaidis A. V., Wikesjö U. M. E. (2006). Biology and principles of periodontal wound healing/regeneration. *Periodontology 2000*.

[2] Bartold P. M., Shi S., Gronthos S. (2006). Stem cells and periodontal regeneration. *Periodontology 2000*.

[3] Padial-Molina M., Marchesan J. T., Taut A. D., Jin Q., Giannobile W. V., Rios H. F. (2012). Methods to validate tooth-supporting regenerative therapies. *Methods in Molecular Biology*.

[4] Padial-Molina M., Rios H. F. (2014). Stem cells, scaffolds and gene therapy for periodontal engineering. *Current Oral Health Reports*.

[5] Karring T., Nyman S., Lindhe J. (1980). Healing following implantation of periodontitis affected roots into bone tissue. *Journal of Clinical Periodontology*.

[6] Nyman S., Karring T., Lindhe J., Planten S. (1980). Healing following implantation of periodontitis-affected roots into gingival connective tissue. *Journal of Clinical Periodontology*.

[7] Nyman S., Lindhe J., Karring T., Rylander H. (1982). New attachment following surgical treatment of human periodontal disease. *Journal of Clinical Periodontology*.

[8] Sculean A., Nikolidakis D., Nikou G., Ivanovic A., Chapple I. L., Stavropoulos A. (2015). Biomaterials for promoting periodontal regeneration in human intrabony defects: a systematic review. *Periodontology 2000*.

[9] Dereka X. E., Markopoulou C. E., Vrotsos I. A. (2006). Role of growth factors on periodontal repair. *Growth Factors*.

[10] Lynch S. E., Williams R. C., Polson A. M. (1989). A combination of platelet-derived and insulin-like growth factors enhances periodontal regeneration. *Journal of Clinical Periodontology*.

[11] Rönnstrand L., Heldin C.-H. (2001). Mechanisms of platelet-derived growth factor-induced chemotaxis. *International Journal of Cancer*.

[12] Lynch S. E., Wisner-Lynch L., Nevins M., Nevins M. L. (2006). A new era in periodontal and periimplant regeneration: use of growth-factor enhanced matrices incorporating rhPDGF. *Compendium of Continuing Education in Dentistry*.

[13] Lynch S. E., Ruiz de Castilla G., Williams R. C. (1991). The effects of short-term application of a combination of platelet-derived and insulin-like growth factors on periodontal wound healing. *Journal of Periodontology*.

[14] Lynch S. E., Buser D., Hernandez R. A. (1991). Effects of the platelet-derived growth factor/insulin-like growth factor-I combination on bone regeneration around titanium dental implants. Results of a pilot study in beagle dogs. *Journal of Periodontology*.

[15] Nevins M. L., Reynolds M. A., Camelo M., Schupbach P., Kim D. M., Nevins M. (2014). Recombinant human platelet-derived growth factor BB for reconstruction of human large extraction site defects. *The International Journal of Periodontics & Restorative*.

[16] Nevins M., Kao R. T., McGuire M. K. (2013). Platelet-derived growth factor promotes periodontal regeneration in localized osseous defects: 36-month extension results from a randomized, controlled, double-masked clinical trial. *Journal of Periodontology*.

[17] Simion M., Rocchietta I., Kim D., Nevins M., Fiorellini J. (2006). Vertical ridge augmentation by means of deproteinized bovine bone block and recombinant human platelet-derived growth factor-BB: a histologic study in a dog model. *International Journal of Periodontics and Restorative Dentistry*.

[18] Schwarz F., Sager M., Ferrari D., Mihatovic I., Becker J. (2009). Influence of recombinant human platelet-derived growth factor on lateral ridge augmentation using biphasic calcium phosphate and guided bone regeneration: a histomorphometric study in dogs. *Journal of Periodontology*.

[19] Froum S. J., Froum S. H., Rosen P. S. (2012). Successful management of peri-implantitis with a regenerative approach: a consecutive series of 51 treated implants with 3- to 7.5-year follow-up. *The International Journal of Periodontics & Restorative Dentistry*.

[20] McGuire M. K., Scheyer E. T., Snyder M. B. (2014). Evaluation of recession defects treated with coronally advanced flaps and either recombinant human platelet-derived growth factor-BB plus *β*-tricalcium phosphate or connective tissue: comparison of clinical parameters at 5 years. *Journal of Periodontology*.

[21] Nevins M., Garber D., Hanratty J. J. (2009). Human histologic evaluation of anorganic bovine bone mineral combined with recombinant human platelet-derived growth factor BB in maxillary sinus augmentation: case series study. *The International Journal of Periodontics & Restorative Dentistry*.

[22] Wallace S., Snyder M., Prasad H. (2013). Postextraction ridge preservation and augmentation with mineralized allograft with or without recombinant human platelet-derived growth factor BB (rhPDGF-BB): a consecutive case series. *International Journal of Periodontics and Restorative Dentistry*.

[23] Simmer J. P., Fincham A. G. (1995). Molecular mechanisms of dental enamel formation. *Critical Reviews in Oral Biology and Medicine*.

[24] Lindskog S., Hammarstrom L. (1982). Formation of intermediate cementum. III: 3H-tryptophan and 3H-proline uptake into the epithelial root sheath of Hertwig in vitro. *Journal of Craniofacial Genetics and Developmental Biology*.

[25] Mellonig J. T. (1999). Enamel matrix derivative for periodontal reconstructive surgery: technique and clinical and histologic case report. *International Journal of Periodontics and Restorative Dentistry*.

[26] Wu S.-M., Chiu H.-C., Chin Y.-T. (2014). Effects of enamel matrix derivative on the proliferation and osteogenic differentiation of human gingival mesenchymal stem cells. *Stem Cell Research and Therapy*.

[27] Schwartz Z., Carnes D. L., Pulliam R. (2000). Porcine fetal enamel matrix derivative stimulates proliferation but not differentiation of pre-osteoblastic 2T9 cells, inhibits proliferation and stimulates differentiation of osteoblast-like MG63 cells, and increases proliferation and differentiation of normal human osteoblast NHOst cells. *Journal of Periodontology*.

[28] Yan X. Z., Rathe F., Gilissen C. (2014). The effect of enamel matrix derivative (Emdogain) on gene expression profiles of human primary alveolar bone cells. *Journal of tissue engineering and regenerative medicine*.

[29] Tonetti M. S., Lang N. P., Cortellini P. (2002). Enamel matrix proteins in the regenerative therapy of deep intrabony defects: a multicentre randomized controlled clinical trial. *Journal of Clinical Periodontology*.

[30] Koop R., Merheb J., Quirynen M. (2012). Periodontal regeneration with enamel matrix derivative in reconstructive periodontal therapy: a systematic review. *Journal of Periodontology*.

[31] Siciliano V. I., Andreuccetti G., Siciliano A. I., Blasi A., Sculean A., Salvi G. E. (2011). Clinical outcomes after treatment of non-contained intrabony defects with enamel matrix derivative or guided tissue regeneration: a 12-month randomized controlled clinical trial. *Journal of Periodontology*.

[32] Queiroz L. A., Santamaria M., Casati M., Silverio K., Nociti-Junior F., Sallum E. (2015). Enamel matrix protein derivative plus synthetic bone substitute for the treatment of mandibular class II furcation defects: a case series. *Quintessence International*.

[33] Jepsen S., Heinz B., Jepsen K. (2004). A randomized clinical trial comparing enamel matrix derivative and membrane treatment of buccal Class II furcation involvement in mandibular molars. Part I: study design and results for primary outcomes. *Journal of Periodontology*.

[34] Döri F., Arweiler N. B., Szántó E., Ágics A., Gera I., Sculean A. (2013). Ten-year results following treatment of intrabony defects with an enamel matrix protein derivative combined with either a natural bone mineral or a *β*-tricalcium phosphate. *Journal of Periodontology*.

[35] Castellanos A. T., de la Rosa M. R., de la Garza M., Caffesse R. G. (2006). Enamel matrix derivative and coronal flaps to cover marginal tissue recessions. *Journal of Periodontology*.

[36] Modica F., Del Pizzo M., Roccuzzo M., Romagnoli R. (2000). Coronally advanced flap for the treatment of buccal gingival recessions with and without enamel matrix derivative. A split-mouth study. *Journal of Periodontology*.

[37] McGuire M. K., Scheyer E. T., Nunn M. (2012). Evaluation of human recession defects treated with coronally advanced flaps and either enamel matrix derivative or connective tissue: comparison of clinical parameters at 10 years. *Journal of Periodontology*.

[38] Chambrone L., Tatakis D. N. (2015). Periodontal soft tissue root coverage procedures: a systematic review from the AAP regeneration workshop. *Journal of Periodontology*.

[39] Favato M. N., Vidigal B. C. L., Cosso M. G., Manzi F. R., Shibli J. A., Zenóbio E. G. (2014). Impact of human maxillary sinus volume on grafts dimensional changes used in maxillary sinus augmentation: a multislice tomographic study. *Clinical Oral Implants Research*.

[40] Miron R. J., Oates C. J., Molenberg A., Dard M., Hamilton D. W. (2010). The effect of enamel matrix proteins on the spreading, proliferation and differentiation of osteoblasts cultured on titanium surfaces. *Biomaterials*.

[41] Boyapati L., Wang H.-L. (2006). The role of platelet-rich plasma in sinus augmentation: a critical review. *Implant Dentistry*.

[42] Shepherd N., Greenwell H., Hill M., Vidal R., Scheetz J. P. (2009). Root coverage using acellular dermal matrix and comparing a coronally positioned tunnel with and without platelet-rich plasma: a pilot study in humans. *Journal of Periodontology*.

[43] Khairy N. M., Shendy E. E., Askar N. A., El-Rouby D. H. (2013). Effect of platelet rich plasma on bone regeneration in maxillary sinus augmentation (randomized clinical trial). *International Journal of Oral and Maxillofacial Surgery*.

[44] Kawase T., Kamiya M., Kobayashi M. (2015). The heat-compression technique for the conversion of platelet-rich fibrin preparation to a barrier membrane with a reduced rate of biodegradation. *Journal of Biomedical Materials Research B: Applied Biomaterials*.

[45] Döri F., Kovács V., Arweiler N. B. (2009). Effect of platelet-rich plasma on the healing of intrabony defects treated with an anorganic bovine bone mineral: a pilot study. *Journal of Periodontology*.

[46] Fernández-Barbero J. E., Galindo-Moreno P., Ávila-Ortiz G., Caba O., Sánchez-Fernández E., Wang H.-L. (2006). Flow cytometric and morphological characterization of platelet-rich plasma gel. *Clinical Oral Implants Research*.

[47] Murakami S. (2011). Periodontal tissue regeneration by signaling molecule(s): what role does basic fibroblast growth factor (FGF-2) have in periodontal therapy?. *Periodontology 2000*.

[48] Mayahara H., Ito T., Nagai H. (1993). In vivo stimulation of endosteal bone formation by basic fibroblast growth factor in rats. *Growth Factors*.

[49] Nakamura T., Hanada K., Tamura M. (1995). Stimulation of endosteal bone formation by systemic injections of recombinant basic fibroblast growth factor in rats. *Endocrinology*.

[50] Murakami S., Takayama S., Kitamura M. (2003). Recombinant human basic fibroblast growth factor (bFGF) stimulates periodontal regeneration in class II furcation defects created in beagle dogs. *Journal of Periodontal Research*.

[51] Takayama S., Murakami S., Shimabukuro Y., Kitamura M., Okada H. (2001). Periodontal regeneration by FGF-2 (bFGF) in primate models. *Journal of Dental Research*.

[52] Cao R., Bråkenhielm E., Pawliuk R. (2003). Angiogenic synergism, vascular stability and improvement of hind-limb ischemia by a combination of PDGF-BB and FGF-2. *Nature Medicine*.

[53] Kao R. T., Murakami S., Beirne O. R. (2009). The use of biologic mediators and tissue engineering in dentistry. *Periodontology 2000*.

[54] Kitamura M., Nakashima K., Kowashi Y. (2008). Periodontal tissue regeneration using fibroblast growth factor -2: randomized controlled phase II clinical trial. *PLoS ONE*.

[55] Kitamura M., Akamatsu M., Machigashira M. (2011). FGF-2 stimulates periodontal regeneration: results of a multi-center randomized clinical trial. *Journal of Dental Research*.

[56] Khoshkam V., Chan H. L., Lin G. H. (2015). Outcomes of regenerative treatment with rhPDGF-BB and rhFGF-2 for periodontal intra-bony defects: a systematic review and meta-analysis. *Journal of Clinical Periodontology*.

[57] Urist M. R. (1965). Bone: formation by autoinduction. *Science*.

[58] Kobayashi M., Takiguchi T., Suzuki R., et al (1999). Recombinant human bone morphogenetic protein-2 stimulates osteoblastic differentiation in cells isolated from human periodontal ligament. *Journal of Dental Research*.

[59] Ripamonti U., Renton L. (2006). Bone morphogenetic proteins and the induction of periodontal tissue regeneration. *Periodontology 2000*.

[60] Arosarena O., Collins W. (2005). Comparison of BMP-2 and -4 for rat mandibular bone regeneration at various doses. *Orthodontics & Craniofacial Research*.

[61] Ripamonti U., Heliotis M., Ferretti C. (2007). Bone morphogenetic proteins and the induction of bone formation: from laboratory to patients. *Oral and Maxillofacial Surgery Clinics of North America*.

[62] Ripamonti U., Dix-Peek T., Parak R., Milner B., Duarte R. (2015). Profiling bone morphogenetic proteins and transforming growth factor-betas by hTGF-beta3 pre-treated coral-derived macroporous bioreactors: the power of one. *Biomaterials*.

[63] Giannobile W. V., Ryan S., Shih M.-S., Su D. L., Kaplan P. L., Chan T. C. K. (1998). Recombinant human osteogenic protein-1 (OP-1) stimulates periodontal wound healing in class III furcation defects. *Journal of Periodontology*.

[64] Wikesjö U. M. E., Guglielmoni P., Promsudthi A. (1999). Periodontal repair in dogs: effect of rhBMP-2 concentration on regeneration of alveolar bone and periodontal attachment. *Journal of Clinical Periodontology*.

[65] Urist M. R. (2002). Bone: formation by autoinduction. 1965. *Clinical Orthopaedics and Related Research*.

[66] Boyne P. J., Marx R. E., Nevins M. (1997). A feasibility study evaluating rhBMP-2/absorbable collagen sponge for maxillary sinus floor augmentation. *International Journal of Periodontics and Restorative Dentistry*.

[67] Triplett R. G., Nevins M., Marx R. E. (2009). Pivotal, randomized, parallel evaluation of recombinant human bone morphogenetic protein-2/absorbable collagen sponge and autogenous bone graft for maxillary sinus floor augmentation. *Journal of Oral and Maxillofacial Surgery*.

[68] Kao D. W. K., Kubota A., Nevins M., Fiorellini J. P. (2012). The negative effect of combining rhBMP-2 and Bio-Oss on bone formation for maxillary sinus augmentation. *The International Journal of Periodontics & Restorative Dentistry*.

[69] Torrecillas-Martinez L., Monje A., Pikos M. A. (2013). Effect of rhBMP-2 upon maxillary sinus augmentation: a comprehensive review. *Implant Dentistry*.

[70] Howell T. H., Fiorellini J., Jones A. (1997). A feasibility study evaluating rhBMP-2/absorbable collagen sponge device for local alveolar ridge preservation or augmentation. *International Journal of Periodontics and Restorative Dentistry*.

[71] Cochran D. L., Jones A. A., Lilly L. C., Fiorellini J. P., Howell H. (2000). Evaluation of recombinant human bone morphogenetic protein-2 in oral applications including the use of endosseous implants: 3-year results of a pilot study in humans. *Journal of Periodontology*.

[72] Fiorellini J. P., Buser D., Riley E., Howell T. H. (2001). Effect on bone healing of bone morphogenetic protein placed in combination with endosseous implants: a pilot study in beagle dogs. *International Journal of Periodontics and Restorative Dentistry*.

[73] de Freitas R. M., Spin-Neto R., Marcantonio Junior E., Pereira L. A. V. D., Wikesjö U. M., Susin C. (2015). Alveolar ridge and maxillary sinus augmentation using rhBMP-2: a systematic review. *Clinical Implant Dentistry and Related Research*.

[74] Ortega-Oller I., Padial-Molina M., Galindo-Moreno P., O'Valle F., Jodar-Reyes A. B., Peula-Garcia J. M. Bone regeneration from PLGA micro-nanoparticles. *BioMed Research International*.

[75] van den Bergh J. P. A., ten Bruggenkate C. M., Groeneveld H. H. J., Burger E. H., Tuinzing D. B. (2000). Recombinant human bone morphogenetic protein-7 in maxillary sinus floor elevation surgery in 3 patients compared to autogenous bone grafts. A clinical pilot study. *Journal of Clinical Periodontology*.

[76] Corinaldesi G., Piersanti L., Piattelli A., Iezzi G., Pieri F., Marchetti C. (2013). Augmentation of the floor of the maxillary sinus with recombinant human bone morphogenetic protein-7: a pilot radiological and histological study in humans. *British Journal of Oral and Maxillofacial Surgery*.

[77] Kraus D., Jäger A., Abuduwali N., Deschner J., Lossdörfer S. (2012). Intermittent PTH(1-34) signals through protein kinase A to regulate osteoprotegerin production in human periodontal ligament cells *in vitro*. *Clinical Oral Investigations*.

[78] Lossdörfer S., Götz W., Jäger A. (2005). PTH(1-34) affects osteoprotegerin production in human PDL cells in vitro. *Journal of Dental Research*.

[79] Lossdörfer S., Götz W., Jäger A. (2006). Parathyroid hormone modifies human periodontal ligament cell proliferation and survival in vitro. *Journal of Periodontal Research*.

[80] Kawane T., Takahashi S., Saitoh H., Okamoto H., Kubodera N., Horiuchi N. (2002). Anabolic effects of recombinant human parathyroid hormone (1–84) and synthetic human parathyroid hormone (1–34) on the mandibles of osteopenic ovariectomized rats with maxillary molar extraction. *Hormone and Metabolic Research*.

[81] Marques M. R., Dias da Silva M. A., Manzi F. R., Cesar-Neto J. B., Nociti F. H., Barros S. P. (2005). Effect of intermittent PTH administration in the periodontitis-associated bone loss in ovariectomized rats. *Archives of Oral Biology*.

[82] Chan H. L., McCauley L. K. (2013). Parathyroid hormone applications in the craniofacial skeleton. *Journal of Dental Research*.

[83] Bashutski J. D., Eber R. M., Kinney J. S. (2010). Teriparatide and osseous regeneration in the oral cavity. *The New England Journal of Medicine*.

[84] Kuchler U., Luvizuto E. R., Tangl S., Watzek G., Gruber R. (2011). Short-term teriparatide delivery and osseointegration: a clinical feasibility study. *Journal of Dental Research*.

[85] Lee J., Wikesjö U. M. E. (2014). Growth/differentiation factor-5: pre-clinical and clinical evaluations of periodontal regeneration and alveolar augmentation—review. *Journal of Clinical Periodontology*.

[86] Kakudo N., Wang Y. B., Miyake S., Kushida S., Kusumoto K. (2007). Analysis of osteochondro-induction using growth and differentiation factor-5 in rat muscle. *Life Sciences*.

[87] Windisch P., Stavropoulos A., Molnár B. (2012). A phase IIa randomized controlled pilot study evaluating the safety and clinical outcomes following the use of rhGDF-5/*β*-TCP in regenerative periodontal therapy. *Clinical Oral Investigations*.

[88] Koch F. P., Becker J., Terheyden H., Capsius B., Wagner W. (2010). A prospective, randomized pilot study on the safety and efficacy of recombinant human growth and differentiation factor-5 coated onto beta-tricalcium phosphate for sinus lift augmentation. *Clinical Oral Implants Research*.

[89] Brockmeyer P., Lange K., Hahn W., Schliephake H., Matthias Gruber R. (2014). Increase of homogenous new bone formation using osteoinductive factor rhGDF-5 during sinus floor augmentation in Goettingen Minipigs. *Clinical Oral Implants Research*.

[90] Schwarz F., Rothamel D., Herten M., Ferrari D., Sager M., Becker J. (2008). Lateral ridge augmentation using particulated or block bone substitutes biocoated with rhGDF-5 and rhBMP-2: an immunohistochemical study in dogs. *Clinical Oral Implants Research*.

[91] Pilipchuk S. P., Plonka A. B., Monje A. (2015). Tissue engineering for bone regeneration and osseointegration in the oral cavity. *Dental Materials*.

[92] Lin Z., Navarro V. P., Kempeinen K. M. (2010). LMP1 regulates periodontal ligament progenitor cell proliferation and differentiation. *Bone*.

[93] Padial-Molina M., Volk S. L., Rios H. F. (2014). Periostin increases migration and proliferation of human periodontal ligament fibroblasts challenged by tumor necrosis factor -*α* and *Porphyromonas gingivalis* lipopolysaccharides. *Journal of Periodontal Research*.

[94] Rosselli-Murai L. K., Almeida L. O., Zagni C. (2013). Periostin responds to mechanical stress and tension by activating the MTOR signaling pathway. *PLoS ONE*.

[95] Lin Z., Rios H. F., Park C.-H. (2013). LIM domain protein-3 (LMP3) cooperates with BMP7 to promote tissue regeneration by ligament progenitor cells. *Gene Therapy*.

[96] Padial-Molina M., Rodriguez J. C., Volk S. L., Rios H. F. (2015). Standardized in vivo model for studying novel regenerative approaches for multitissue bone–ligament interfaces. *Nature Protocols*.

